# Inhibition of NRF2 enhances the acute myeloid leukemia cell death induced by venetoclax via the ferroptosis pathway

**DOI:** 10.1038/s41420-024-01800-2

**Published:** 2024-01-18

**Authors:** Xibao Yu, Yan Wang, Jiaxiong Tan, Yuchen Li, Pengyue Yang, Xuan Liu, Jing Lai, Yue Zhang, Letong Cai, Yinfeng Gu, Ling Xu, Yangqiu Li

**Affiliations:** 1 The First Affiliated Hospital and Institute of Hematology, School of Medicine, Jinan University, Guangzhou, 510632 China; 2 Key Laboratory for Regenerative Medicine of Ministry of Education, Jinan University, Guangzhou, 510632 China; 3 Guangzhou Municipality Tianhe Nuoya Bio-engineering Co. Ltd, Guangzhou, 510663 China; 4 Department of Pediatric Oncology, Tianjin Medical University Cancer Institute and Hospital, National Clinical Research Center for Cancer, Key Laboratory of Cancer Prevention & Therapy of Tianjin, Tianjin, 300060 China

**Keywords:** Cell death, Cancer therapy

## Abstract

Venetoclax, an inhibitor that selectively targets B cell lymphoma-2 (BCL-2) that has been approved for treating adult acute myeloid leukemia (AML) in combination with hypomethylating agents. However, its short duration of response and emergence of resistance are significant issues. In this study, we found that the sensitivity of AML cells to venetoclax was considerably enhanced by ML385, an inhibitor of the ferroptosis factor nuclear transcription factor erythroid 2-related factor 2 (NRF2). Using AML samples, we verified that NRF2 and its target gene ferritin heavy chain 1 (FTH1) were highly expressed in patients with AML and correlated with poor prognosis. Downregulation of NRF2 could inhibit FTH1 expression and significantly enhance the venetoclax-induced labile iron pool and lipid peroxidation. By contrast, NRF2 overexpression or administration of the reactive oxygen species inhibitor N-acetylcysteine and vitamin E could effectively suppress the anti-AML effects of ML385+venetoclax. Furthermore, the ferroptosis inducer erastin increased the anti-AML effects of venetoclax. Our study demonstrated that NRF2 inhibition could enhance the AML cell death induced by venetoclax via the ferroptosis pathway. Thus, the combination of ML385 with venetoclax may offer a favorable strategy for AML treatment.

## Introduction

Acute myeloid leukemia (AML) is a malignancy that arises from the stem cell precursors of the lineage of myeloid cells. Chromosomal abnormalities and/or gene mutations usually drive the differentiation failure and proliferation advantage of hematopoietic precursors, subsequently impeding normal hematopoiesis [1]. With the substantially enhanced understanding of AML pathogenesis, B cell lymphoma-2 (BCL-2), FMS-like tyrosine kinase 3 (FLT3), isocitrate dehydrogenases types 1 and 2 (IDH1/2) inhibitors, and other targeted therapeutic drugs have found utility in the clinical management of patients with AML and special genetic alterations [2–7]. However, the long-term overall survival (OS) of high-risk patients remains unsatisfactory; in particular, the 5-year survival rate of elderly patients is less than 20% [3, 4]. Thus, developing new therapeutics for AML is imperative.

Venetoclax, the first and only US Food and Drug Administration (FDA)-approved BCL-2 selective inhibitor, competes with Bim to bind to BCL-2, disrupting the formation of the BCL-2-Bim complex and activating the mitochondrial apoptosis pathway [8]. The FDA granted approval to Venetoclax for the treatment of patients with chronic lymphocytic leukemia and older AML. Notably, in patients with newly diagnosed AML are elderly or have complications unsuitable for induction chemotherapy, BCL-2 inhibitors combined with low-dose cytarabine (Ara-C) or hypomethylating agents (HMAs), such as azacitidine and decitabine, can improve the survival of older or unfit patients. However, resistance to BCL-2 inhibitors in AML is a major cause of treatment failure [9, 10]. Therefore, the mechanism underlying the resistance to these inhibitors must be studied, and new synergistic treatments should be developed.

Nuclear transcription factor erythroid 2-related factor 2 (NRF2) is a crucial regulatory factor of the antioxidant response, and its constitutive activation can promote the occurrence of various tumors and increase anti-tumor drug resistance [11, 12]. For example, the expression of the oncogenes Myc, Braf, and Kras can directly promote NRF2 transcription and initiate the antioxidant program, reducing intracellular reactive oxygen species (ROS) levels, enhancing cell protection and anabolism, and finally promoting tumor progression and drug resistance [13, 14]. In addition, epigenetic modifications and mutations in NRF2 and Kelch-like ECH-associated protein 1 (Keap1) can activate NRF2, which confers drug resistance [15–19]. Ectopic expression of NRF2 can clear ROS by up-regulating antioxidant enzymes [for example, glutamate-cysteine ligase catalytic subunit (GCLC), heme oxygenase-1 and NADH quinone oxidoreductase 1] and help cancer cells escape death; by contrast, downregulating NRF2 expression in tumor cells can promote the killing induced by therapeutic drugs [20]. Therefore, targeted inhibition of NRF2 has potential in tumor treatment, but the role of NRF2 inhibitors in AML treatment is unclear.

Ferroptosis is an iron- and ROS-dependent form of cell death, leading to the massive buildup of lipid ROS [21]. In recent studies, targeting ferroptosis opens new avenues for the development of anti-cancer therapy. Many critical ferroptosis proteins, such as solute carrier family 7 member 11 (SLC7A11, the subunit of the cystine/glutamate antiporter xCT), ferritin heavy chain 1 (FTH1, the heavy chains of ferritin), and GCLC/ glutamate–cysteine ligase modifier subunit (GCLM, which control the level of GSH), are well-defined NRF2 target genes [22–25]. NRF2 is known to be a negative regulator of ferroptosis [25]. However, its underlying mechanisms of ferroptotic regulation in AML are not fully understood.

Our previous study found that promyelocytic leukemia (PML)/retinoic acid receptor-alpha (RARα) can maintain the nuclear expression levels of NRF2 by inhibiting the degradation rate of the NRF2 protein, and inhibition of the NRF2 signaling pathway makes acute promyelocytic leukemia (APL) cells more sensitive to arsenic trioxide (ATO) and Ara-C [26]. Therefore, targeted inhibition of NRF2 has the potential to enhance the sensitivity of AML cells to chemotherapeutic agents and even other targeted therapeutic agents. The mechanism of venetoclax-induced cell death also involves the generation of free radicals, inducing ROS accumulation and glutathione depletion [27, 28]. Thus, the combination of an NRF2 inhibitor with venetoclax may be an efficient way to enhance leukemia inhibition in AML.

Here, we explored the anti-leukemia efficiency of the NRF2-specific inhibitor ML385 together with venetoclax in AML cell lines and elucidated the mechanism of their synergistic effects, which may be due to activation of the ferroptosis pathway.

## Results

### Overexpression of NRF2 is associated with poor prognosis for patients with AML

**Fig. 1:**
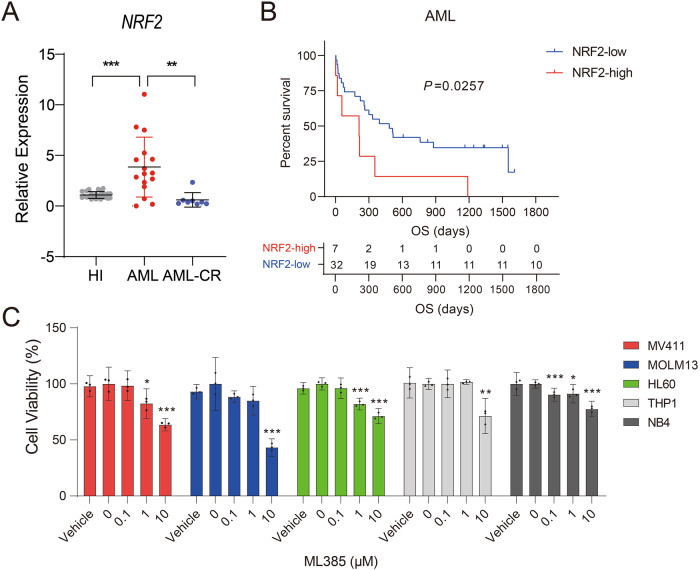
NRF2 is highly expressed in AML patients and associated with poor prognosis. **A** Comparison of *NRF2* expression by qRT-PCR. The expression of *NRF2* was examined in PBMCs from healthy donors (*n* = 16) and primary AML (*n* = 25) and AML-CR (*n* = 8) patients. *ACTB* was used as a housekeeping gene for normalization. Each data point represents one patient sample. **B** Kaplan–Meier curves were plotted using AML patients with high or low NRF2 expression (*n* = 39). **C** Viability of the AML cell lines MV411, MOLM13, HL60, THP1, and NB4 that were treated with ML385 at corresponding concentrations for 48 h. Data are shown as the mean ± 95% CI. *P < 0.05, **P < 0.01, ***P < 0.001 (**A**, **C**: one-way ANOVA with Bonferroni post hoc test).

We previously showed that *NRF2* was overexpressed in APL [26]. In this study, we examined the mRNA level of *NRF2* in PBMCs from patients with newly diagnosed AML (patients without APL). Consistent with previous results, *NRF2* expression was significantly elevated compared with that in healthy individuals (HIs) and AML patients who achieved complete remission (AML-CR; Fig. 1A). Moreover, patients with high *NRF2* expression had inferior survival rates (cut-off value was calculated using X-tile software; Fig. 1B). To determine whether *NRF2* overexpression influences the survival of AML cells, we treated five types of AML cell lines (MV411, MOLM13, HL60, THP1, and NB4) with ML385, a specific inhibitor of NRF2. We observed that ML385 could significantly inhibit the growth of above cell lines in a dose-dependent manner (Fig. 1C). The above results indicated that NRF2 activation was associated with AML cell survival.

### Inhibition of NRF2 enhances the sensitivity of AML cells to venetoclax

**Fig. 2:**
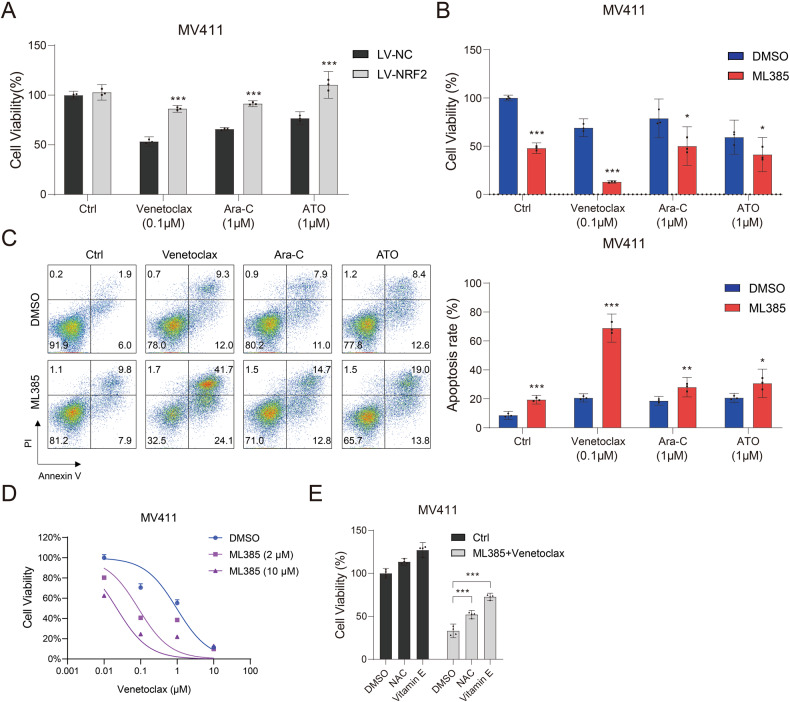
Inhibition of NRF2 can enhance the sensitivity of AML cells to venetoclax. **A** The viability of MV411-LV-NRF2 and MV411-LV-NC cells treated with venetoclax (0.1 μM), Ara-C (1 μM), or ATO (1 μM) for 48 h. **B** The viability and (**C**) apoptosis of MV411 cells treated with ML385 (10 μM) alone or in combination with venetoclax (0.1 μM), Ara-C (1 μM), or ATO (1 μM) for 48 h. **D** The viability of MV411 cells treated with ML385 and venetoclax at different concentrations for 48 h. **E** Viability of MV411 cells pretreated with 5 mM NAC or vitamin E for 4 h followed by treatment with the combination of venetoclax (0.1 μM) and ML385 (10 μM) for 48 h. Experiments were performed in triplicate, and the mean ± 95% CI from three independent experiments was plotted. Error bars indicate standard deviation. *P < 0.05, **P < 0.01, ***P < 0.001 (**A**–**C**, **E**: one-way ANOVA with Bonferroni post hoc test).

We have previously shown that NRF2 inhibition boosts the sensitivity of APL cells to ATO and Ara-C [26]. In this research, we constructed an MV411 cell line that stably overexpressed NRF2 (Fig. S1) and further found that NRF2 overexpression made the cell line resistant not only to ATO and Ara-C but also venetoclax (Fig. 2A). By contrast, the NRF2 inhibitor ML385 could significantly promote the sensitivity of MV411 cells to ATO, Ara-C, and venetoclax (Fig. 2B). Notably, the apoptosis of MV411 cells induced by ML385 combined with venetoclax was more significant than that induced by the combination with ATO or Ara-C (Fig. 2C). In addition, ML385 (10 μM) acted synergistically with venetoclax (0.1 μM) to induce AML cell death (combinational index = 0.08417; Fig. 2D and Supplementary Table 1). This synergistic effect was further confirmed in two additional AML cell lines (Fig. S2). In addition, the anti-AML effects of ML385+ venetoclax could be inhibited by the ROS inhibitors N-acetylcysteine (NAC) and Vitamin E (Fig. 2E).

The combination of venetoclax with HMAs has been demonstrated to improve outcomes for older or unfit patients with AML [9]. However, retrospective and prospective studies have shown that combinations of venetoclax and HMAs are passive in relapsed or refractory AML (R/R-AML) treatment [29–31]. Here, we demonstrated that treatment with the combination of venetoclax and ML385 induced similar (in MOLM13) or even increased levels (in MV411) of cell death in AML cell lines, compared with treatment with venetoclax and azacitidine or decitabine (Fig. S3). These results indicated that the combination of ML385 and venetoclax induced significantly greater cell death than treatment with venetoclax and HMAs. Thus, this kind of combination may have a potential in treating R/R-AML.

### Overexpression of NRF2 enriches the ferroptosis pathway

**Fig. 3:**
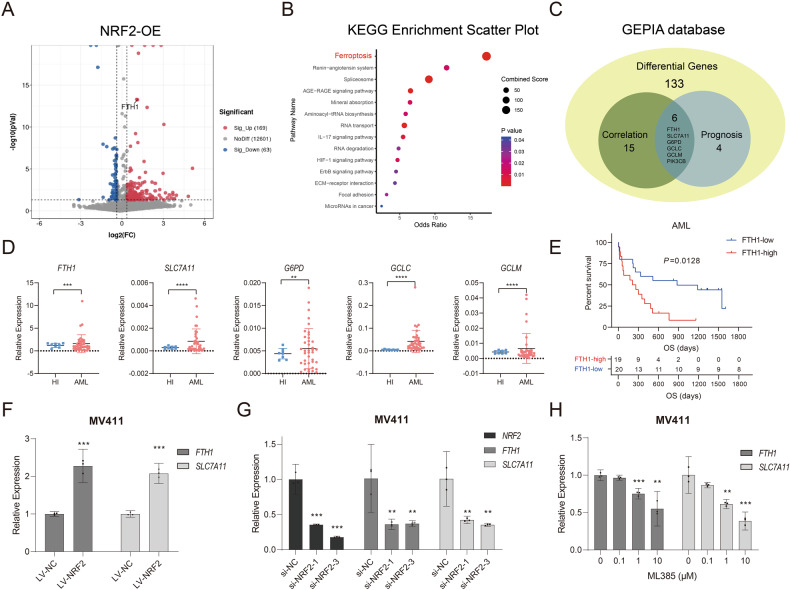
Overexpression of NRF2 can enrich the ferroptosis pathway. **A** Volcano and (**B**) KEGG enrichment plots comparing the differential expression of mRNAs in NB4-LV-NRF2 cells relative to NB4-LV-NC cells. **C** Intersection between AML prognostic genes and genes that are highly correlated with NRF2 expression in the GEPIA database and differentially expressed genes in cells with NRF2 overexpression. **D** Comparison of *FTH1, SLC7A11, G6PD, GCLC*, and *GCLM* expression in PBMCs from HIs (*n* = 8) compared with AML patients (*n* = 38). **E** Kaplan–Meier curves were plotted using AML patients with high or low FTH1 expression (*n* = 39). **F**–**H** qRT–PCR analysis of NRF2, *FTH1*, and *SLC7A11* expression in MV411-LV-NRF2 or MV411 cells after knocking down NRF2 or MV411 cells treated with ML385 at corresponding concentrations. Data are shown as the mean ± 95% CI. *P < 0.05, **P < 0.01, ***P < 0.001 (**D**, **F**: two-tailed unpaired Student’s *t*-tests; **G**, **H** one-way ANOVA with Bonferroni post hoc test).

NRF2 is involved in the oxidative stress reaction, but it also participates in other signal transduction pathways. To better understand the potential NRF2 regulatory mechanism in AML, we analyzed transcriptome sequencing data from NB4 cells that overexpressed NRF2 (NRF2-OE) and found that the differentially expressed genes in NRF2-OE cells were enriched for the ferroptosis pathway (Fig. 3A, B). Using the Gene Expression Profiling Interactive Analysis (GEPIA) database, we found that the ferroptosis-related genes *FTH1*, *SLC7A11*, *G6PD*, *GCLC, GCLM*, and *PIK3CB* were associated with the prognosis of patients with AML (Fig. S4). In addition, the expression of these genes was positively correlated with NRF2 expression and dysregulated in NRF2-OE cells (Fig. 3C). We then performed qPCR assays to verify the gene expression levels of *FTH1, SLC7A11, G6PD, GCLC*, and *GCLM* in our AML cohorts. The data demonstrated that five of these genes were overexpressed in our AML samples, (Fig. 3D) but no significant difference was found in *PIK3CB* expression (data not shown). High expression of FTH1 (cut-off values was calculated using X-tile software) was found to be associated with poor prognosis for patients with AML (Fig. 3E), thereby suggesting that NRF2/FTH1 was relevant to the occurrence and progression of AML. Furthermore, the use of the MV411 cell line that stably overexpressed NRF2 verified that NRF2 overexpression could promote the expression of FTH1 and SLC7A11 (Fig. 3F). By contrast, NRF2-siRNA or the NRF2-specific inhibitor ML385 could inhibit the expression of FTH1 and SLC7A11 (Fig. 3G,H). These results suggested that NRF2 could regulate FTH1 and SLC7A11 expression and was favorable for AML cell survival.

### ML385 sensitizes AML cells to the cell death induced by venetoclax via activation of the ferroptosis pathway

**Fig. 4:**
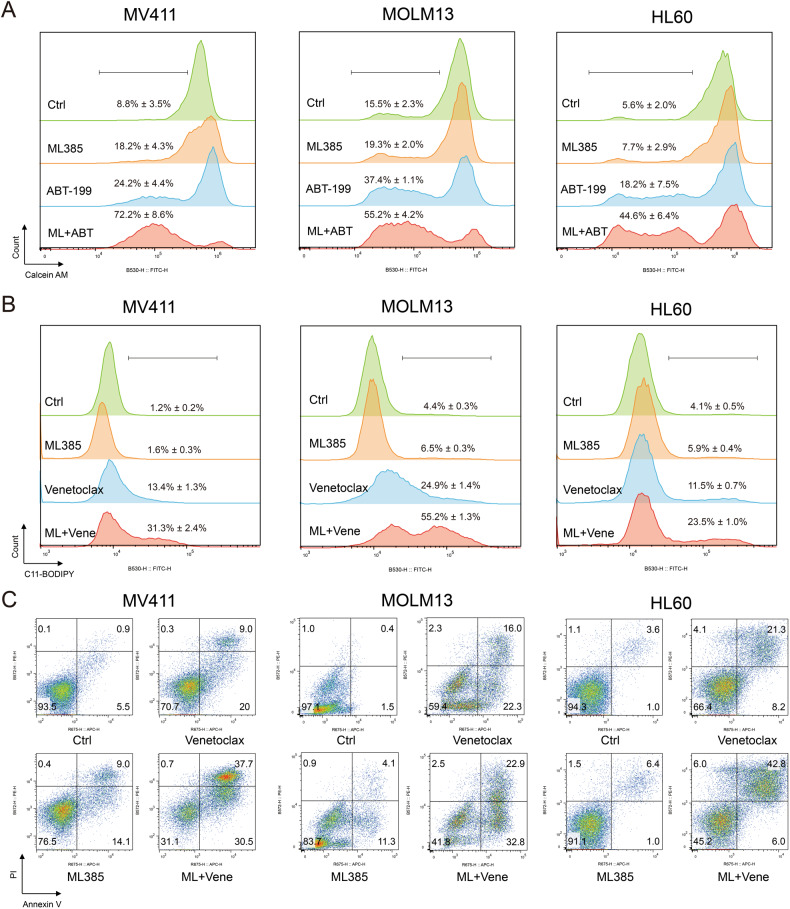
ML385 promotes ferroptosis in AML cells induced by venetoclax. **A** The levels of the intracellular labile iron pool, **B** lipid ROS, and **C** apoptosis of MV411 (left), MOLM13 (middle) and HL60 (right) cells treated with venetoclax (0.1 μM), ML385 (10 μM), or the combination of venetoclax and ML385 for 48 h. Experiments were performed in triplicate, and data are shown as the mean ± 95% CI. ML ML385. Vene, Venetoclax.

**Fig. 5:**
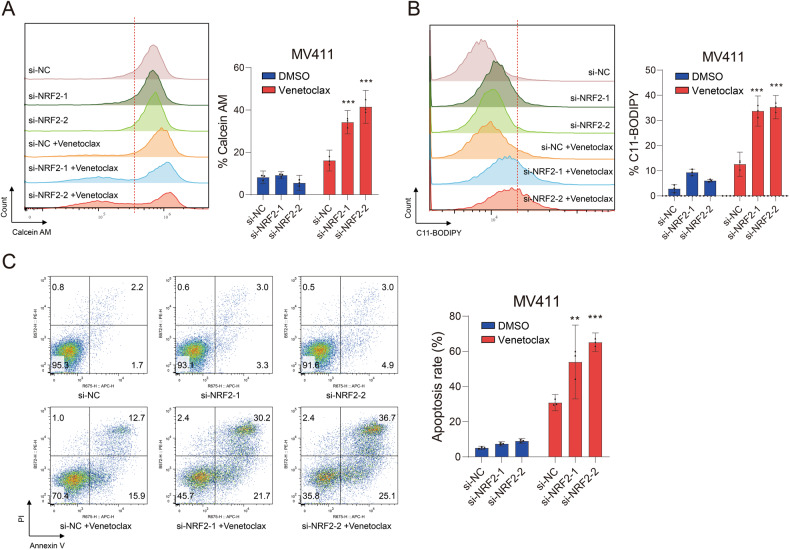
Inhibition of NRF2 promotes the ferroptosis induced by venetoclax in AML cells. **A** The levels of the intracellular labile iron pool, **B** lipid ROS, and (**C**) apoptosis of MV411 cells after knocking down NRF2. Experiments were performed in triplicate, and the mean from three independent experiments was plotted. Error bars indicate 95% CI. *P < 0.05, **P < 0.01, ***P < 0.001. **A**–**C** one-way ANOVA with Bonferroni post hoc test).

The above results indicated that ML385 may sensitize AML cells to the cell death induced by venetoclax via activation of the ferroptosis pathway. Thus, we further analyzed the cell death; the levels of the intracellular labile iron pool; and the lipid peroxidation of the AML cell lines MV411, MOLM13, and HL60 induced by the combination of ML385 and venetoclax. The results demonstrated that the percentage of cell death for all three cell lines increased with the simultaneous application of ML385 and venetoclax. This result was accompanied with increased levels of the intracellular labile iron pool and lipid ROS (Fig. 4). Similarly, down-regulation of NRF2 expression by siRNA could promote an increase in the labile iron pool, lipid ROS, and apoptosis induced by venetoclax in MV411 cells (Fig. 5). These results suggested that the susceptibility of AML cells to venetoclax could be enhanced by inhibiting NRF2 through activation of the ferroptosis pathway.

### Ferroptosis inducer erastin enhanced the anti-AML effects of venetoclax

**Fig. 6:**
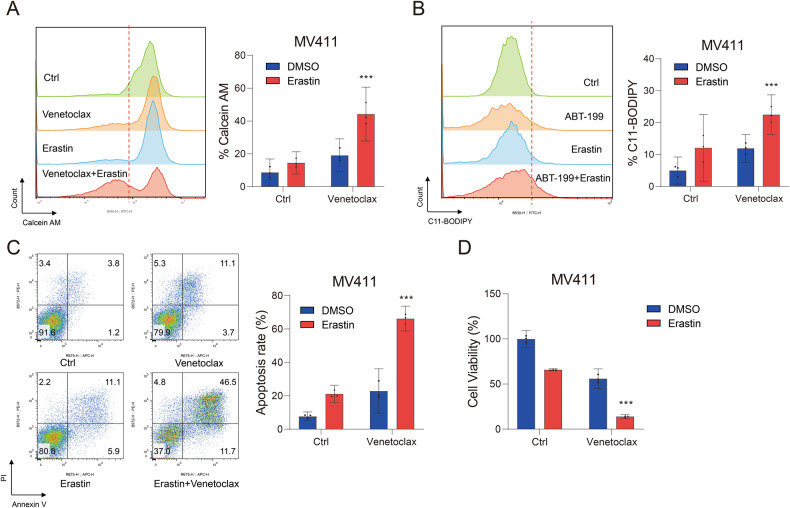
Ferroptosis inducer Erastin enhanced the anti-AML effect of venetoclax. **A** The levels of intracellular the labile iron pool, (**B**) lipid ROS, (**C**) apoptosis, and (**D**) cell viability of MV411 cells treated with venetoclax (0.1 μM), erastin (10 μM), or the combination of venetoclax and erastin for 48 h. Experiments were performed in triplicate, and the mean from three independent experiments was plotted. Error bars indicate 95% CI. *P < 0.05, **P < 0.01, ***P < 0.001. (**A**–**D**: one-way ANOVA with Bonferroni post hoc test).

Given that inhibition of NRF2 can raise the sensitivity of AML cells to venetoclax through ferroptosis, we wondered if ferroptosis inducers had the same effect. To validate this hypothesis, we combined erastin, a cystine transporter inhibitor that induces ferroptotic cell death, and venetoclax to treat MV411 cells. We found that erastin could also promote the level of the labile iron pool, lipid ROS, and apoptosis in MV411 cells induced by venetoclax (Fig. 6A–C), and the combination of erastin and venetoclax significantly inhibited the viability of MV411 cells (Fig. 6D). Thus, these results demonstrated that erastin increased the susceptibility of AML cells to venetoclax, which was similar to the effects of ML385.

## Discussion

In this work, we observed for the first time that the expression of NRF2 was correlated with AML survival, and the sensitivity of AML cells to venetoclax was considerably enhanced by the NRF2 inhibitor ML385.

The widespread utilization of venetoclax has introduced a new challenge of resistant, while in patients with R/R-AML or high-risk classification, venetoclax treatment showed no response [32, 33]. Developing venetoclax-based combination therapies is one strategy for overcoming resistance and expand the scope of agent application. Recent studies have shown that combining venetoclax with small-molecule inhibitors (such as FLT3 inhibitors, MCL-1 inhibitors, and cyclin-dependent kinase 9 inhibitors) has a synergistic effect in the treatment of high-risk AML [34–37]. Notably, we found that the NRF2 inhibitor ML385 could considerably improve the sensitivity of AML cells to venetoclax, and this effect was better than that of venetoclax combined with HMAs. This paradigm provides a highly attractive therapeutic strategy for combining NRF2 inhibitors and venetoclax for the treatment of AML. Mechanistically, NRF2 may make AML cells more sensitive to BCL-2 inhibitors via activation of the ferroptosis pathway.

NRF2 is an essential regulator of the cellular antioxidant response; it can combine with antioxidant reaction elements in DNA to regulate the expression of antioxidant proteins and protect cells from oxidative stress damage [11, 38]. Constitutive activation of NRF2 can promote the occurrence of various tumors and increase tumor drug resistance [11, 12]. Thus, NRF2 inhibition has remarkable potential in tumor treatment [39, 40]. Studies have reported that the NRF2 signaling pathway is highly triggered in AML [41–43], while NRF2 pathway inhibition improves the sensitivity of AML cells to daunorubicin and Ara-C [43–46]. FTH1 and SLC7A11 are key genes in regulating ferroptosis. Recently, it has been reported that NRF2 can inhibit ferroptosis by activating the expression of FTH1 and SLC7A11, respectively, then inhibiting the labile iron pool and promoting the synthesis of glutathione [47–51]. Notably, Lei Feng et al. found that SLC7A11 deletion in NRF2-OE KYSE 150 cell lines restored the lipid peroxidation levels and cell death induced by radiotherapy [47]. In agreement with prior findings, we found that the expression levels of NRF2 mRNA and FTH1, SLC7A11 were drastically increased in samples from patient with primary AML compared with those in HIs and patients with AML-CR. Patients with a higher NRF2 expression level had a lower survival rate compared to those with a lower NRF2 expression level. For the first time, we found that use of the NRF2 antagonist ML385 (ML385 is an innovative and selective NRF2 inhibitor that can specifically bind the Neh1 domain of NRF2, inhibiting downstream target gene expression) could induce the apoptosis of AML cells. More importantly, ML385 can significantly and synergistically enhance the anti-AML effects of venetoclax. Previously, NRF2 inhibitors such as valproic acid, ATRA, brusatol, and luteolin have been reported to induce the apoptosis of AML cells alone or in combination with other agents [46, 52–54]. As reported by Cheng C et al., combining Ara-C with brusatol increases the sensitivity of AML cell lines to Ara-C; however, we found more elevated cell death when combining ML385 with venetoclax compared with Ara-C or ATO.

Mechanistically, we found that venetoclax treatment alone induced the labile iron pool and lipid ROS; however, inhibition of NRF2 expression suppressed FTH1 expression and greatly augmented the venetoclax-induced labile iron pool and lipid ROS. Our findings suggested that NRF2 might augment the responsiveness of AML cells to BCL-2 inhibitors through activation of the ferroptosis pathway. Additionally, the use of erastin, a ferroptosis inducer, could also improve the sensitivity of AML cells to venetoclax, which was similar to the effects of ML385. Although venetoclax induces apoptosis in cancer cells by stimulating the mitochondrial apoptosis pathway, other mechanisms could not be ruled out. Ferroptosis is a novel programmed cell death pathway that is dependent on iron and ROS. Indeed, significant advancements in recent years have enhanced our comprehension of the role of ferroptosis in the initiation and progression of various types of tumors. However, the role of ferroptosis in AML is still not fully understood. A previous study indicated that the administration of low-dose erastin promoted the death of HL-60 cells induced by Ara-C and doxorubicin [55]. Here, our data demonstrated that erastin could enhance the anti-AML effects of venetoclax in MV4-11 cells. Thus, targeting the ferroptosis pathway may be a promising avenue for AML treatment, particularly in synergism with other agents.

The results presented here are supportive but not conclusive, so further investigations are warranted. First, the molecular mechanism involved in the regulation of the ferroptosis pathway by NRF2 is not yet fully elucidated. Second, although the synergistic effect of ML385 and venetoclax has been validated in vitro, the synergistic effect in vivo remains to be experimentally confirmed.

**Fig. 7:**
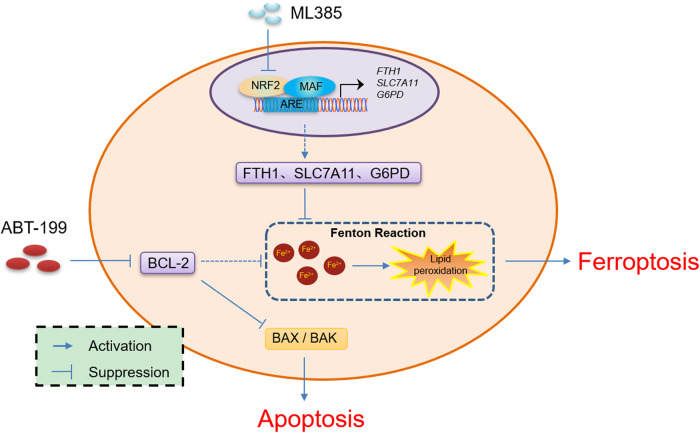
Proposed model depicting the regulation and role of venetoclax/ML385 in AML. Inhibition of NRF2 enhances the sensitivity of AML cells to BCL-2 inhibitors through promoting an increase in the labile iron pool, lipid ROS, and apoptosis.

Overall, we observed that inhibition of NRF2 boosted the sensitivity of AML cells to BCL-2 inhibitors through the ferroptosis pathway (Fig. 7). Thus, the combination of ML385 with venetoclax may offer a favorable strategy for AML treatment.

## Materials and methods

### Clinical samples

Peripheral blood mononuclear cells (PBMCs) from 88 patients with de novo AML and 25 HIs were analyzed in this study. The clinical information of AML and HIs was described in our previous work [26, 56]. Prior consent was obtained for all samples included in this study, and this study received approval from the Ethics Committee of the Affiliated Hospitals of Jinan University.

### Cultured cells and inhibitors

The human AML cell lines MV4-11, MOLM13, HL60, THP-1, and NB4 were grown in RPMI 1640 medium supplemented with 10% fetal bovine serum. All cells were generated at 37 °C in a humidified atmosphere with 5% CO_2_. Venetoclax and ML385 were procured from Selleck Chemicals (Shanghai, China).

### Generation of stable cell lines

To ectopically express NRF2, a lentivirus overexpressing NRF2 (LV-NRF2) and a control virus (LV-NC) were obtained from OBiO Technology. The MV4-11 cells were exposed to lentiviruses with a multiplicity of infection (MOI) of 30. Stable pools were selected by treating with 5 μg/mL puromycin for a duration of 5 days.

### RNA interference

The Neon Transfection System (Invitrogen, CA, USA) was employed to transfect MV4-11 cells with 100 pmol of small interfering RNAs (siRNAs), following the established protocol [26, 57]. The siRNAs were procured from RiboBio (Guangzhou, China), with the siRNA sequences being provided in Supplementary Table 2.

### RNA extraction and Quantitative real-time PCR

Total RNA was extracted using TRIzol reagent (Invitrogen, CA, USA), and the High-Capacity cDNA Reverse Transcription Kits (Applied Biosystems, CA, USA) were employed to synthesize first-strand cDNA, following the manufacturer’s instructions [58]. Quantitative real-time PCR (qRT-PCR) was performed using SYBR Green and *ACTB* was used as an internal control. The primer sequences can be found in Supplementary Table 3. The qRT-PCR cycling program consisted of an initial step at 95 °C for 15 min, followed by 40 cycles of denaturation at 95 °C for 10 s and annealing/extension at 60 °C for 30 s.

### Calcein AM and C11-BODIPY staining

To evaluate the level of ferroptosis, target cells were cultured with Calcein AM (1 μM, Abcam, MA, USA) or C11-BODIPY (2.5 μM, Abclonal, Wuhan, China) according to the manufacturers’ instructions.

### Apoptosis assays

After the respective treatments, cells were washed with chilled PBS. Subsequently, they were stained using the Annexin V/PI Apoptosis Kit (MultiSciences, Hangzhou, China). Flow cytometry analysis was conducted in conformity with the manufacturer’s instructions to assess apoptosis levels.

### Cell proliferation assays

The Cell Counting Kit-8 (CCK-8, Biosharp, China) was utilized to evaluate cell viability, following the manufacturer’s instructions. Initially, AML cells were seeded at a concentration of 5000 cells per well and cultured in RPMI 1640 medium supplemented with 10% FBS along with the corresponding drugs for different time points. At the end of each experiment, the CCK-8 reagent was added to the wells. Following incubation at 37 °C for 2 h, the absorbance of each well was measured at 450 nm using a microplate reader. A blank control without cells was used to account for background absorbance. After calculations, cell viability was normalized to the respective controls. The combinational index for drug combinations was calculated through CompuSyn software, and combinational index values < 1 indicate drug synergy.

### Statistical analysis

Data are presented as the mean ± 95% confidence intervals (CI). All experiments were conducted with three or more biological replicates per experimental group. Statistical analysis was performed using SPSS 22.0 software. The statistical significance of differences between groups was assessed using the Student’s *t* test (unpaired and two-tailed) or one-way ANOVA with Bonferroni post hoc test for multiple comparisons. A significance level of less than 0.05 (*P* < 0.05) was considered statistically significant. *P < 0.05, **P < 0.01, ***P < 0.001, and ns no significance.

### Supplementary information

Supplementary Material- clean version

## Data Availability

The datasets used and/or analyzed during the current study are available from the corresponding author upon reasonable request.
