# Supplementary material for: Inhibition of NRF2 enhances the acute myeloid leukemia cell death induced by venetoclax via the ferroptosis pathway
Source: Cell Death Discov. 2024 Jan 18;10:35. doi: 10.1038/s41420-024-01800-2 (PMC10796764; doi:10.1038/s41420-024-01800-2)
Supplement: Supplementary file 1 — Supplementary Material- clean version [file 41420_2024_1800_MOESM1_ESM.docx]

**Supplementary Material**

**Supplementary Table 1:** Combinational Index

| Venetoclax (μM) | ML385 (μM) | Effect | CI |
| --- | --- | --- | --- |
| 0.01 | 2.0 | 0.8 | 1.05449 |
| 0.1 | 2.0 | 0.4 | 0.11333 |
| 1.0 | 2.0 | 0.47 | 1.07477 |
| 10.0 | 2.0 | 0.1 | 0.98523 |
| 0.01 | 10.0 | 0.62 | 1.01995 |
| 0.1 | 10.0 | 0.24 | 0.08417 |
| 1.0 | 10.0 | 0.21 | 0.29987 |
| 10.0 | 10.0 | 0.12 | 1.24890 |

**Supplementary Table 2:** siRNA and shRNA sequences

| Gene target | Sense (5' -> 3') | Antisense (5' -> 3') | |
| --- | --- | --- | --- |
| si-NRF2-1 | AAGAGUAUGAGCUGGAAAAAC | | GUUUUUCCAGCUCAUACUCUU |
| si-NRF2-2 | GCUUUUGGCGCAGACAUUC | | GAAUGUCUGCGCCAAAAGC |
| si-NC | siN05815122147 | |  |

**Supplementary Table 3:** Primer sequences

| Gene target | Sense (5' -> 3') | Antisense (5' -> 3') | |
| --- | --- | --- | --- |
| *ACTB* | TTGTTACAGGAAGTCCCTTGCC | | ATGCTATCACCTCCCCTGTGTG |
| *NRF2* | GAGAGCCCAGTCTTCATTGC | | TTGGCTTCTGGACTTGGAAC |
| *FTH1* | AATTTTCCGCAAGGTTCGATT | | ACTCTTTCAGTGGTGGG |
| *SLC7A11* | GAGTGTGGCCTAGAGCTGG | | GGCTCAGTCCTGATAGCAGTG |
| *G6PD* | CCTCGTGCTGTCGGACCCATA | | CAGGCTTGTGCTCTGCTTGTGA |
| *GCLC* | TCTTCATTGACCAAGGAAATCGG | | TCCGGGGTGCATTATCTCTAC |
| *GCLM* | CAGCTTGACTCAAAATTCCTGGA | | TGAAGATTACGCTTGCTTTTCCT |

**Supplementary Figures**


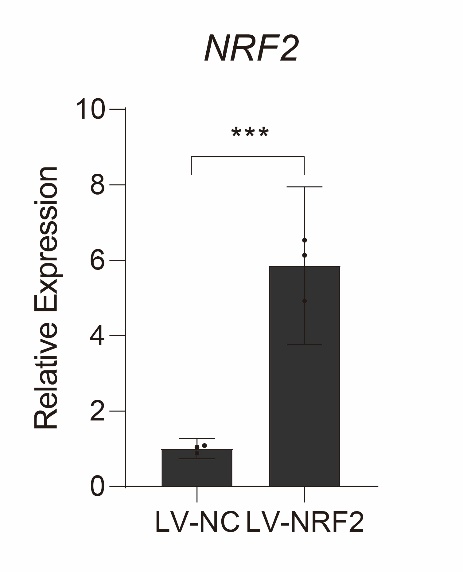


**Supplementary Figure 1. The MV411 cell line stably overexpressing NRF2 was constructed**

qRT–PCR analysis of NRF2 in MV411-LV-NRF2. Data are shown as the mean ± 95% CI. The significance of differences between groups was determined by the Student’s *t* test (unpaired and two-tailed). *, *P* < 0.05, **, *P* < 0.01, ***, *P* < 0.001, and ns, no significance.


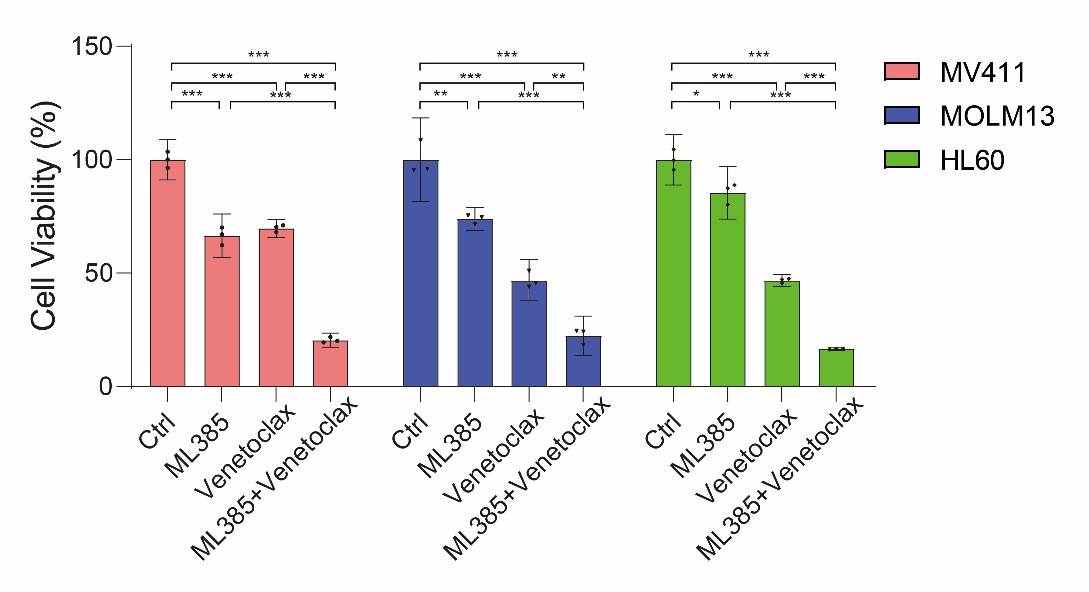
**Supplementary Figure 2. ML385 promotes cell death in AML cells induced by venetoclax**

The cell viability of MV411 (left), MOLM13 (middle) and HL60 (right) cells treated with venetoclax (0.1 μM), ML385 (10 μM), or the combination of venetoclax and ML385 for 48 h. Experiments were performed in triplicate, and the mean from three independent experiments was plotted. Error bars indicate 95% CI. *, *P*<0.05, **, *P*<0.01, ***, *P*<0.001. (one-way ANOVA with Bonferroni post hoc test).


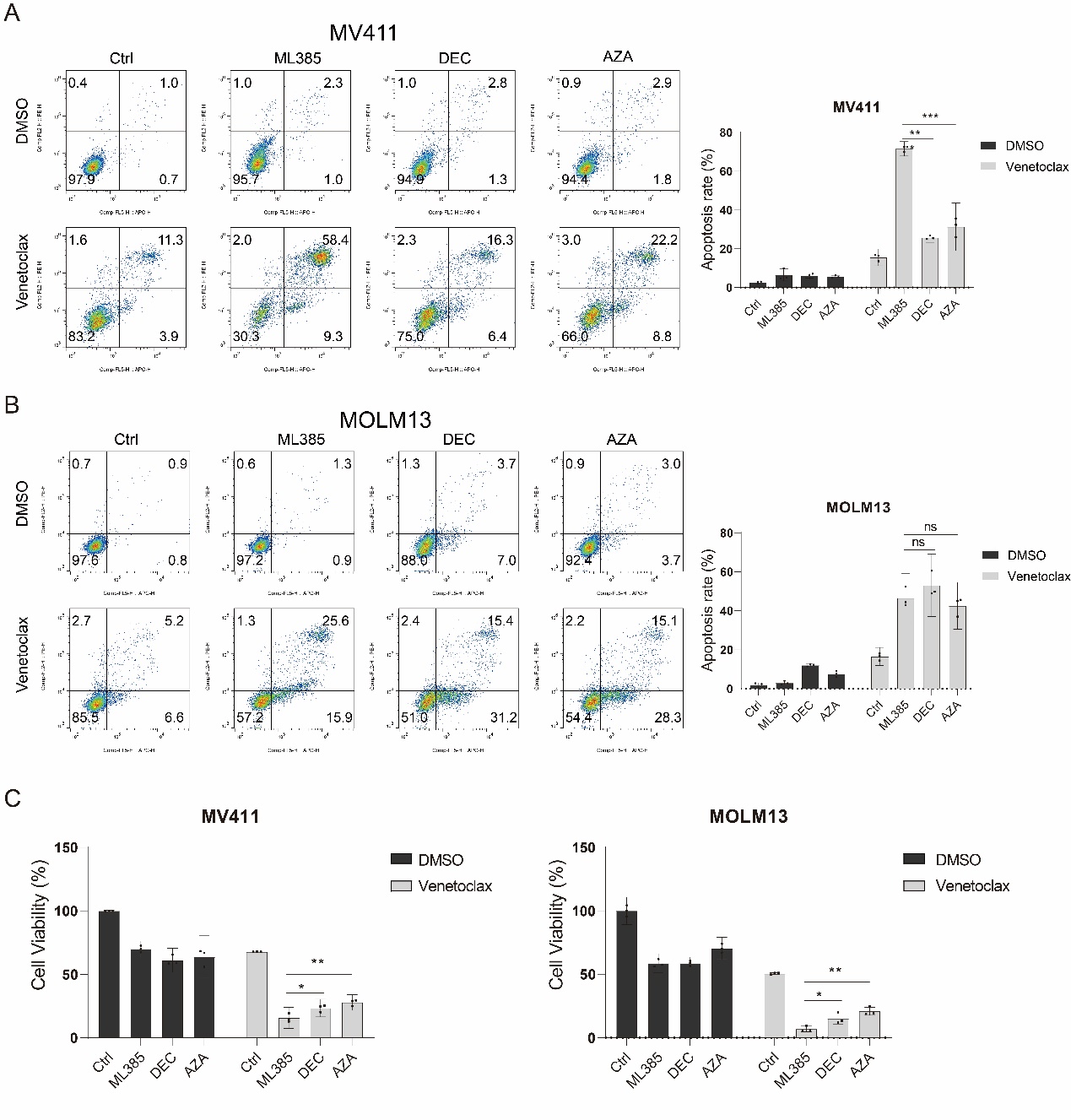


**Supplementary Figure 3. The combination of ML385 and venetoclax induces significantly greater cell death when compared with treatment with venetoclax and HMAs**

(A-B) The apoptosis rate of MV411 (A) and MOLM13 (B) cells treated with ML385 (10 μM), decitabine (10 μM), azacitidine (1 μM) or the combination of venetoclax (0.1 μM) for 48 h. (C) The cell viability of MV411 (left) and MOLM13 (right) cells treated with ML385, decitabine, azacitidine or the combination of venetoclax for 48 h. Experiments were performed in triplicate, and the mean from three independent experiments was plotted. Error bars indicate 95% CI. *, *P*<0.05, **, *P*<0.01, ***, *P*<0.001. (A-D: one-way ANOVA with Bonferroni post hoc test). DEC, decitabine; AZA, azacitidine.


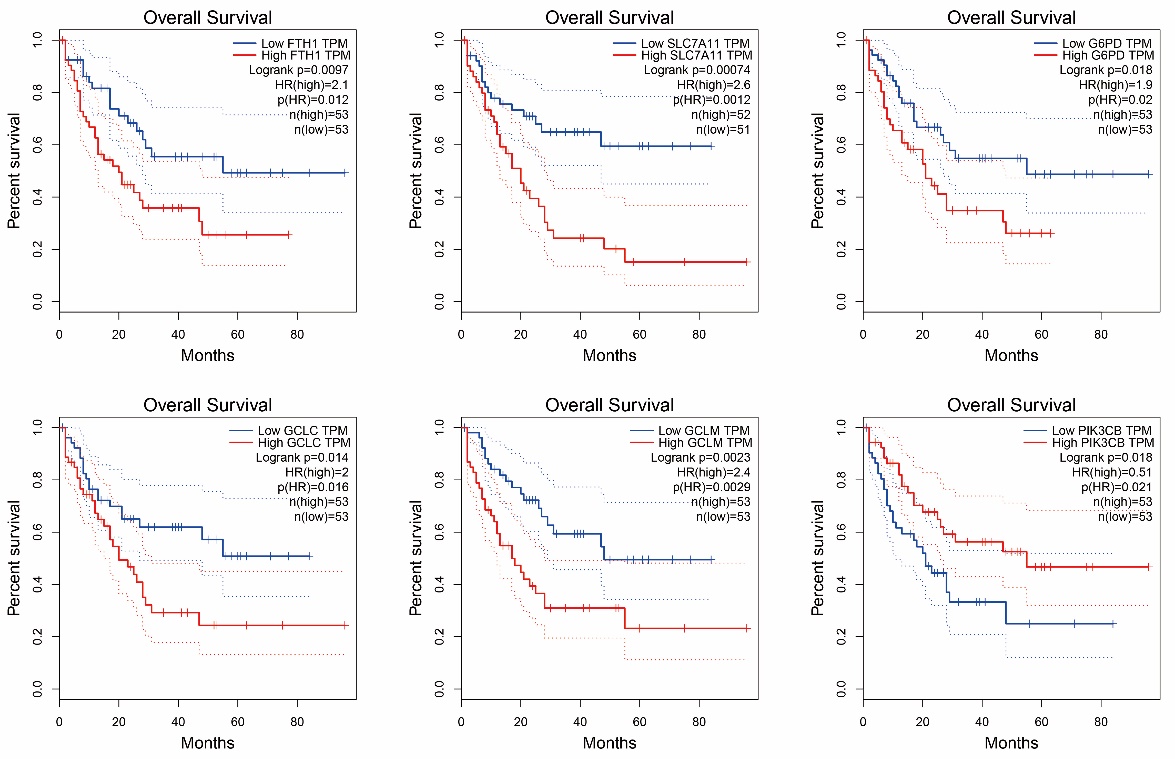


**Supplementary Figure 4. The ferroptosis-related genes *FTH1, SLC7A11, G6PD, GCLC, GCLM*, and *PIK3CB* were associated with the prognosis of AML patients**

High expression of *FTH1, SLC7A11, G6PD, GCLC, GCLM* were correlated with poor OS in AML patients; Low expression of *PIK3CB* were correlated with poor OS in AML patients
